# GSTM1 and GSTT1 polymorphisms associated with pain in a chemotherapy-induced peripheral neuropathy cohort

**DOI:** 10.1007/s00432-023-04677-3

**Published:** 2023-03-20

**Authors:** Paul J. Dunn, Lyn R. Griffiths, Patsy Yates, Larisa M. Haupt, Kim E. Alexander

**Affiliations:** 1 Centre for Genomics and Personalised Health, Genomics Research Centre, School of Biomedical Sciences, Queensland University of Technology (QUT), 60 Musk Ave., Brisbane, QLD 4059 Australia; 2 ARC Training Centre for Cell and Tissue Engineering Technologies, Queensland University of Technology (QUT), Brisbane, QLD 4059 Australia; 3 Max Planck Queensland Centre for the Materials Sciences of Extracellular Matrices, Brisbane, QLD 4059 Australia; 4 School of Medicine, Faculty of Health Sciences and Medicine, Bond University, Gold Coast, QLD 4227 Australia; 5 School of Nursing, Faculty of Health, Queensland University of Technology (QUT), Victoria Park Road, Brisbane, QLD 4059 Australia

**Keywords:** GSTM1, GSTT1, GSTP1, Chemotherapy induced peripheral neuropathy, Pain

## Abstract

**Purpose:**

Chemotherapy induced peripheral neuropathy (CIPN) is a debilitating condition that is a direct consequence of receiving cancer treatment. The molecular aetiology of CIPN is not well understood, and it is theorised that there may be a genetic component. Genetic polymorphisms in Glutathione-S Transferase (GST) genes, including *GSTT1, GSTM1* and *GSTP1,* encode for enzymes known to metabolise drugs used in chemotherapy, and have been theorised to be associated with CIPN. This study aimed to investigate four markers in these genes for an association in a mixed cancer cohort in relation to CIPN (*n* = 172).

**Methods:**

CIPN was measured using the neuropathy item from the Patient Reported Outcome Common Terminology Criteria for Adverse Event (PRO-CTCAE) assessment. Genotyping for all samples was performed using PCR for the *GSTM1* and *GSTT1* null variants and restriction fragment length polymorphisms for the *GSTP1* and *GSTM1* polymorphisms.

**Results:**

No associations were found for the GST gene markers in relation to CIPN within our study, or CIPN severity. Longitudinal stratification of the CIPN phenotypes to examine links for neuropathy, identified nominally significant protective associations with the GSTM* null allele (*p*-value = 0.038, OR = 0.55) and the presence of pain at month 2 of treatment, as well as a risk factor for pain related month 2 of treatment for individuals with the GSTT1*null allele (*p*-value = 0.030, OR = 1.64). Higher severity of pain in patients with CIPN persisted at each time-point compared to those without CIPN.

**Conclusion:**

No significant results for an association between CIPN with polymorphisms in GSTM1, GSTT1 and GSTP1 were identified. However, associations for the GSTM1¬-null and GSTT1-null polymorphisms with pain at month 2 following chemotherapy were identified.

**Supplementary Information:**

The online version contains supplementary material available at 10.1007/s00432-023-04677-3.

## Introduction/background

Chemotherapy induced peripheral neuropathy (CIPN) is a condition causing debilitating nerve pain, numbness or tingling due to exposure to common chemotherapy treatments used for cancer. CIPN is predominantly a sensory neuropathy that may be accompanied by motor and autonomic changes. It is similar to other neuropathic pain conditions in that it can be stimulus dependent or independent (Woolf and Mannion 1999). Between 19 and 85% of patients receiving chemotherapy will experience CIPN, which can be accompanied by fatigue, distress and decline in physical function. Furthermore, at least 30% of these individuals are unable to return to normal health and function (Fallon 2013). Currently there are no preventative treatments or management strategies for CIPN with established effectiveness, with the most common management strategy aimed at reducing or stopping chemotherapy (Hershman et al. 2014; Seretny et al. 2014).

While there have been several studies completed on identifying risk factors for CIPN, the pathophysiological causes are not well understood and epidemiological understanding is currently limited (Mizrahi et al. 2021; Molassiotis et al. 2019; Seretny et al. 2014). CIPN is thought to be multifactorial and involve microtubule disruption, oxidative stress, and mitochondrial damage, altered ion channel activity, myelin sheath damage, DNA damage, immunological processes and neuroinflammation (Areti et al. 2014). These postulated mechanisms are thought to underpin the sensory phenomena of CIPN and result in axonal hyperexcitability and nociceptor sensitisation. These postulated mechanisms have also led to theories of a genetic aetiology, where genome-wide association studies (GWAS) and candidate gene studies have helped identify candidate genes thought to be associated with CIPN (Chua and Kroetz 2017; Cliff et al. 2017).

One group of genes which have been investigated previously, belong to the group of Glutathione-S Transferases (*GSTs),* which includes *GSTT1, GSTP1* and *GSTM1,* with some studies identifying associations between polymorphic markers in these genes and CIPN (Cliff et al. 2017). Although some of these studies showed promising results, they were not easily replicated in independent cohorts and as such the role that these genetic variations play in CIPN is still debatable (Cliff et al. 2017). Despite the lack of reproducibility, soluble GSTs are known to interact with chemotherapeutic agents such as Adriamycin, 1,3-bis(2-chloroethyl)-1-nitrosourea (BCNU), busulfan, carmustine, chlorambucil, cis-platin, crotonyloxymethyl-2- cyclohexanone (COMC-6), cyclophosphamide, ethacrynic acid, melphalan, mitoxantrone, and thiotepa, and are detoxified by GST (Hayes et al. 2005). Furthermore, both *GSTM1* and *GSTT1* both exhibit a common polymorphism that is characterized by the complete deletion of the gene (null allele) (Strange and Fryer 1999). These null alleles result in the absence of enzyme and its catalytic activity, which has been shown to decrease the cells' ability to detoxify certain genotoxic agents (Norppa 2004). An adenine to guanine transition (rs1695) in *GSTP1* has also been shown to decrease enzymatic activity for this protein. It has been thought that this can alter the pharmacokinetics relating to treatment outcomes and toxicity (Board et al. 1989; Ma et al. 2017). Considering this role, we aimed to investigate *GSTT1, GSTM1* and *GSTP1* characterised polymorphic markers in a mixed cohort of cancer patients receiving chemotherapy.

## Methods

### Participant selection

Participant data used in this study is part of a larger prospective, longitudinal, observational study to assess symptoms that cancer patients experience in the first four months following the initiation of anti-neoplastic agents (*n* = 251). Participants for the study were included if they were > 18 years of age, competent to give informed consent, and received a minimum dosage of exposure to CIPN-causing agents based on the National Comprehensive Cancer Network CIPN Taskforce Report (Dorsey et al. 2019).

### Clinical measurement

Symptom experience was measured using the National Cancer Institute Patient Reported Outcome Common Terminology Criteria for Adverse Event (PRO-CTCAE) tool to document adverse effects in cancer treatment and contains 124 items that reflect 78 cancer treatment related symptoms (Basch et al. 2014; National Cancer Institute 2016). A modified PRO-CTCAE tool enabled participants to self-report the frequency and severity of adverse events and interference with daily activities in the previous month (as opposed to previous week), scoring items on a five-point Likert scale (Basch et al. 2014; Kluetz et al. 2016; National Cancer Institute 2016). The timing of this assessment was made based on the results of a pilot of the tool on 30 participants where completion of the tool on a weekly basis was determined to be too burdensome for participants to consistently complete (data not included in the main study), and previous work that determined not distinctly different results were obtained when using a four week recall period when compared to daily reporting (Mendoza et al. 2017). This study focussed on using the two neuropathy items (“what was the severity of your numbness or tingling in your hands or feet at its worst?” and “how much did numbness or tingling in your hands or feet interfere with your usual activities”) and three pain items (“how often did you have pain?”, “what was the severity of your pain at its worst?”, and “how much did pain interfere with your usual or daily activities”) of this tool as phenotypic markers in this study. Both markers factored in the severity at its worst in the past month and how these symptoms interfered with usual or daily activities. The use of these items in the measurement of CIPN correlate with measurement using other widely used patient-reported measures of CIPN, such as the Quality-of-Life Questionnaire-Chemotherapy-Induced Peripheral Neuropathy Scale (QLQ-CIPN20) (Knoerl et al. 2017).

### Laboratory investigations–DNA extractions and genotyping

Approximately 10–20 mL of blood was collected in EDTA tubes and DNA was extracted using the traditional salting-out method and quantified using a Nanodrop™ 8000 Spectrophotometer (Waltham, Massachusetts). DNA working stocks of 50 µL were made and diluted to a concentration of 20 ng/µL.

Polymorphic markers for *GSTM1* (null variant, and A* and B* polymorphism), *GSTT1* (null variant) and *GSTP1* (rs1695) were genotyped using polymerase chain reaction (PCR) and subsequent restriction enzyme digestion involving either *BsmAI* for GSTP1 and *HaeII* for *GSTM1* A* and B* markers (Supplementary 1). Primers for *GSTM1*, *GSTT1*, β-actin and *GSTP1*-1) and all restriction enzymes were obtained through New England Biolabs and the protocol was based on work completed by Curran et al. (Curran et al. 2000).

### Analysis

Summary statistics for patient data and demographics were generated using the *TableOne* package in R v3.5.1 with p-values disregarded. We first categorised participants according to their worst self-reported level of neuropathy severity into the following categories No CIPN, Mild CIPN, Moderate Severe CIPN, and Very Severe CIPN. Due to the lower distribution of numbers, we combined the Moderate, Severe, and very severe categories to allow for more robust statistical tests to be completed. We then checked the distribution of worst CIPN severity against the patient cancer types and comorbidities (Supplementary 2). We also checked the distribution of pain and neuropathy variables across monthly timepoints against worst CIPN severity. An association test for CIPN (yes/no) against the genotype results for *GSTT1, GSTP1* and *GSTM1* was completed using PLINK v1.9. This included classifying participants as cases if they had any CIPN at any stage during their treatment experience and controls if they exhibited no CIPN. We also investigated the experience of pain and neuropathy across different time-points in relation to the *GST* mutations in the same manner in PLINK v1.9.

## Results

There were originally 189 blood samples received, 17 samples were excluded due to missing data related to chemotherapy treatments and/or CIPN frequency. This resulted in 172 blood samples available for analysis. Participants remaining were comprised of *n* = 107 females (age = 57.4 ± 12.7, min = 29, max = 100), *n* = 62 males (age = 67.3 ± 10.2, min = 35, max = 88), and three participants that did not disclose their gender. The distribution of males and females across each CIPN severity remained similar (within 10% of each other), as such, gender differences were not considered for downstream analyses. The mean age of the cohort was 61.1 ± 12.7, with a similar distribution across all CIPN severities (No CIPN = 62.7 ± 12.0, mild CIPN = 60.5 ± 13.5, moderate/severe CIPN = 60.5 ± 12.7). Out of these participants, n = 142/172 (82.6%) described their ethnicity as European, the remaining participants described a range of ethnicities or did not disclose their ethnicity. From the remaining samples, *n* = 42/172 (24.4%) exhibited no CIPN, *n* = 50/172 (29.1%) showed only mild CIPN, whist *n* = 80/172 (46.5%) participants were classified as having either moderate, severe, or very severe CIPN symptoms at any timepoint (Supplementary 2). Breast cancer was the most common diagnosis in the cohort. High blood pressure was the most common comorbidity reported.

Pain and neuropathy variables at different monthly points were stratified by participant experience of CIPN at its worst (Table 1). By month 4, 76.2% of participants with no CIPN either never or rarely exhibited any pain symptoms, compared to 76.0% and 60.1% for mild-CIPN and moderate-very severe CIPN participants respectively (Table 1). For those that exhibited no CIPN, or Moderate–Severe CIPN, the proportion of individuals suffering from high levels of pain severity decreased each month. Whereas for participants with only mild CIPN, the percentage of participants who experience severe or very severe pain in each month remained constant. The degree at which the pain associated with post-treatment CIPN interfered with normal routines also decreased across all CIPN severity categories with between 57.5 and 94.0% of all participants at any time point found to report that pain had little effect on their ability to complete normal tasks regardless of CIPN severity.

**Table 1:** Longitudinal evaluation of the pain and neuropathy measurements in the cohort based on their worst neuropathy severity rating (No CIPN, mild CIPN or moderate-severe CIPN)

	No CIPN (*n* = 42)				Mild CIPN (*n* = 50)				Moderate-Severe CIPN (*n* = 80)			
	Month 1	Month 2	Month 3	Month 4	Month 1	Month 2	Month 3	Month 4	Month 1	Month 2	Month 3	Month 4
Pain Symptoms Frequency												
Never	10 (23.8%)	18 (42.9%)	23 (54.7%)	21 (50%)	15 (30.0%)	21 (38.0%)	22 (44.0%)	22 (44.0%)	14 (17.4%)	24 (30.0%)	31 (38.8%)	26 (32.6%)
Rarely	13 (31.0%)	13 (31.0%)	6 (14.3%)	11 (26.2%)	15 (30.0%)	11 (22.0%)	15 (30.0%)	16 (32.0%)	17 (21.2%)	17 (21.2%)	17 (21.2%)	22 (27.5%)
Occasionally	12 (28.6%)	9 (21.4%)	5 (11.9%)	5 (11.9%)	13 (26.0%)	13 (26.0%)	2 (4.0%)	6 (12.0%)	30 (37.5%)	21 (26.2%)	9 (11.2%)	17 (21.2%)
Almost Constantly	5 (11.9%)	0 (0.0%)	7 (16.7%)	5 (11.9%)	7 (14.0%)	6 (12.0%)	11 (22.0%)	5 (10.0%)	14 (17.5%)	15 (18.8%)	21 (26.2%)	10 (12.5%)
Frequently	2 (4.8%)	2 (4.8%)	1 (2.4%)	0 (0.0%)	0 (0.0%)	1 (2.0%)	0 (0.0%)	1 (2.0%)	5 (6.2%)	3 (3.8%)	2 (2.5%)	5 (6.2%)
Pain Severity												
None	11 (26.2%)	18 (42.9%)	23 (54.8%)	21 (50.0%)	17 (34.0%)	19 (38.0%)	22 (44.0%)	22 (44.0%)	17 (21.3%)	25 (31.2%)	34 (42.5%)	26 (32.5%)
Mild	14 (33.3%)	11 (26.2%)	6 (14.3%)	13 (31.0%)	16 (32.0%)	18 (36.0%)	20 (40.0%)	16 (32.0%)	19 (23.8%)	17 (21.2%)	18 (22.5%)	26 (32.5%)
Moderate	8 (19.0%)	10 (23.8%)	9 (21.4%)	6 (14.3%)	15 (30.0%)	10 (20.0%)	5 (10.0%)	9 (18.0%)	27 (33.8%)	25 (31.2%)	20 (25.0%)	21 (26.2%)
Severe	8 (19.0%)	2 (4.8%)	3 (7.1%)	2 (4.8%)	1 (2.0%)	3 (6.0%)	3 (6.0%)	3 (6.0%)	14 (17.5%)	10 (12.5%)	7 (8.8%)	5 (6.2%)
Very Severe	1 (2.4%)	1 (2.4%)	1 (2.4%)	0 (0.0%)	1 (2.0%)	0 (0.0%)	0 (0.0%)	0 (0.0%)	3 (3.8%)	3 (3.8%)	1 (1.2%)	2 (2.5%)
Pain Interference												
Not at all	22 (52.4%)	27 (64.3%)	28 (66.7%)	33 (78.6%)	27 (54.0%)	28 (56.0%)	35 (70.0%)	32 (64.0%)	31 (38.7%)	36 (45.0%)	43 (53.8%)	45 (56.3%)
A little bit	8 (19.0%)	8 (19.0%)	9 (21.4%)	4 ( 9.5%)	16 (32.0%)	14 (28.0%)	12 (24.0%)	11 (22.0%)	15 (18.8%)	19 (23.8%)	23 (28.7%)	22 (27.5%)
Somewhat	6 (14.3%)	4 (9.5%)	1 (2.4%)	4 (9.5%)	6 (12.0%)	6 (12.0%)	3 (6.0%)	5 (10.0%)	19 (23.8%)	14 (17.5%)	8 (10.0%)	6 (7.5%)
Quite a bit	5 (11.9%)	2 (4.8%)	4 (9.5%)	1 (2.4%)	1 (2.0%)	2 (4.0%)	0 (0.0%)	2 (4.0%)	12 (15.0%)	10 (12.5%)	5 (6.2%)	6 (7.5%)
Very much	1 (2.4%)	1 (2.4%)	0 (0.0%)	0 (0.0%)	0 (0.0%)	0 (0.0%)	0 (0.0%)	0 (0.0%)	3 (3.8%)	1 (1.2%)	1 (1.2%)	1 (1.2%)
Neuropathy Severity												
None	42 (100.0%)	42 (100.0%)	42 (100.0%)	42 (100.0%)	27 (54.0%)	18 (36.0%)	22 (44.0%)	25 (50.0%)	38 (47.5%)	23(28.8%)	19 (23.7%)	18 (22.6%)
Mild	0 (0.0%)	0 (0.0%)	0 (0.0%)	0 (0.0%)	23 (46.0%)	32 (64.0%)	28 (56.0%)	25 (50.0%)	20 (25.0%)	26 (32.5%)	23 (28.7%)	13 (16.2%)
Moderate	0 (0.0%)	0 (0.0%)	0 (0.0%)	0 (0.0%)	0 (0.0%)	0 (0.0%)	0 (0.0%)	0 (0.0%)	16 (20.0%)	23 (28.7%)	27 (33.8%)	35 (43.8%)
Severe	0 (0.0%)	0 (0.0%)	0 (0.0%)	0 (0.0%)	0 (0.0%)	0 (0.0%)	0 (0.0%)	0 (0.0%)	4 (5.0%)	7 (8.8%)	9 (11.2%)	10 (12.5%)
Very Severe	0 (0.0%)	0 (0.0%)	0 (0.0%)	0 (0.0%)	0 (0.0%)	0 (0.0%)	0 (0.0%)	0 (0.0%)	2 (2.5%)	1 (1.2%)	2 (2.5%)	4 (5.0%)
Neuropathy Interference												
Not at all	42 (100.0%)	42 (100.0%)	42 (100.0%)	42 (100.0%)	46 (92.0%)	44 (88.0%)	44 (88.0%)	42 (84.0%)	55 (68.7%)	46 (57.4%)	35 (43.8%)	39 (46.3%)
A little bit	0 (0.0%)	0 (0.0%)	0 (0.0%)	0 (0.0%)	4 (8.0%)	6 (12.0%)	5 (10.0%)	8 (16.0%)	18 (22.5%)	22 (27.5%)	27 (33.8%)	28 (35.0%)
Somewhat	0 (0.0%)	0 (0.0%)	0 (0.0%)	0 (0.0%)	0 (0.0%)	0 (0.0%)	1 (2.0%)	0 (0.0%)	5 (6.2%)	7 (8.8%)	11 (13.8%)	13 (16.2%)
Quite a bit	0 (0.0%)	0 (0.0%)	0 (0.0%)	0 (0.0%)	0 (0.0%)	0 (0.0%)	0 (0.0%)	0 (0.0%)	1 (1.2%)	4 (5.0%)	5 (6.2%)	9 (11.2%)
Very Much	0 (0.0%)	0 (0.0%)	0 (0.0%)	0 (0.0%)	0 (0.0%)	0 (0.0%)	0 (0.0%)	0 (0.0%)	1 (1.2%)	1 (1.2%)	2 (2.5%)	1 (1.2%)

From *n* = 172 samples there were four samples with missing failed genotyping which were excluded from further study. This included three samples with missing genotypes for *GSTT1* and *n* = 1 sample with missing genotype data for *GSTM1* due to a lack of detection of the β-actin marker. These samples also failed to indicate whether they had CIPN, or what the severity was and were then excluded from any further analysis. Initial analysis completed an association for the GST markers with *n* = 42 (24.4%) samples used as unaffected controls and *n* = 130 samples as affected cases. We did not identify an association with any of the GST markers and CIPN in this cohort, however, the *GSTM1-*null allele was approaching significance with a *p*-value of 0.0617 (Table 2). Longitudinal stratification of the CIPN phenotypes looking at links for neuropathy identified nominally significant protective associations with the GSTM1*null allele (*p*-value = 0.038, OR = 0.55) and the presence of pain at month 2 of treatment, as well as a risk factor for pain related month 2 of treatment for individuals with the GSTT1*null allele (*p*-value = 0.030, OR = 1.64).

**Table 2:** Analysis of major phenotypes associated with CIPN over a period of 4 months of chemotherapy treatment

SNP	Major Allele	Minor Allele	Phenotype	Frequency of Affected	Frequency of Unaffected	CHISQ	*P*-value	Odds Ratio	Standard error	L95	U95
GSTT1 Null	Null Allele	No Del	CIPN (Neuropathy at any timepoint)	0.1966	0.125	2.085	0.1488	1.713	0.376	0.8198	3.579
			Pain Month 1	0.1694	0.1951	0.2824	0.5951	0.841	0.3261	0.4439	1.593
			Pain Month 2	0.1429	0.2333	4.315	0.03778*	0.5476	0.2924	0.3088	0.9713
			Pain Month 3	0.1685	0.1842	0.139	0.7093	0.8977	0.2896	0.5089	1.584
			Pain Month 4	0.1919	0.1515	0.8925	0.3448	1.33	0.3025	0.7352	2.406
			Neuropathy Month 1	0.1935	0.165	0.434	0.51	1.214	0.2948	0.6813	2.164
			Neuropathy Month 2	0.2093	0.1392	2.79	0.09483	1.636	0.2965	0.9151	2.926
			Neuropathy Month 3	0.1765	0.175	0.00123	0.972	1.01	0.2894	0.5729	1.781
			Neuropathy Month 4	0.1928	0.1585	0.6674	0.414	1.268	0.2906	0.7172	2.24
GSTM1 Null	Null Allele	No Del	CIPN (Neuropathy at any timepoint)	0.4417	0.561	3.491	0.06171	0.6191	0.2577	0.3736	1.026
			Pain Month 1	0.4882	0.4524	0.3243	0.569	1.155	0.2526	0.7038	1.894
			Pain Month 2	0.5229	0.4	4.687	0.03039*	1.644	0.2305	1.047	2.583
			Pain Month 3	0.4783	0.4805	0.001714	0.967	0.991	0.2186	0.6456	1.521
			Pain Month 4	0.5	0.4478	0.8843	0.347	1.233	0.2231	0.7964	1.91
			Neuropathy Month 1	0.4219	0.5143	2.721	0.09902	0.6892	0.226	0.4425	1.073
			Neuropathy Month 2	0.4494	0.5125	1.343	0.2466	0.7765	0.2184	0.5061	1.191
			Neuropathy Month 3	0.5	0.4568	0.6311	0.427	1.172	0.2182	0.7754	1.824
			Neuropathy Month 4	0.4767	0.4819	0.009094	0.924	0.9794	0.2178	0.6391	1.501
GSTM1 SNP	B	A	CIPN (Neuropathy at any timepoint)	0.2761	0.2778	0.00039	0.9842	0.9918	0.4193	0.436	2.256
			Pain Month 1	0.2923	0.2609	0.1651	0.6845	1.17	0.3872	0.5479	2.5
			Pain Month 2	0.2885	0.2778	0.02388	0.8772	1.054	0.3407	0.5406	2.055
			Pain Month 3	0.2917	0.275	0.0596	0.8071	1.086	0.3363	0.5615	2.099
			Pain Month 4	0.3039	0.2568	0.4691	0.4934	1.264	0.3423	0.6462	2.472
			Neuropathy Month 1	0.2973	0.2745	0.1095	0.7407	1.118	0.3375	0.577	2.167
			Neuropathy Month 2	0.3061	0.2564	0.5277	0.4676	1.279	0.3395	0.6577	2.489
			Neuropathy Month 3	0.2614	0.3068	0.447	0.5038	0.7994	0.3351	0.4145	1.542
			Neuropathy Month 4	0.2222	0.3488	3.466	0.06263	0.5333	0.3398	0.274	1.038
GSTP1 SNP	G	A	CIPN (Neuropathy at any timepoint)	0.3708	0.439	1.197	0.274	0.7531	0.2596	0.4528	1.253
			Pain Month 1	0.3937	0.369	0.1616	0.6877	1.11	0.26	0.6669	1.848
			Pain Month 2	0.3761	0.4083	0.3378	0.5611	0.8736	0.2325	0.5539	1.378
			Pain Month 3	0.3859	0.3896	0.004943	0.944	0.9844	0.2241	0.6344	1.527
			Pain Month 4	0.3627	0.4254	1.336	0.2477	0.769	0.2275	0.4924	1.201
			Neuropathy Month 1	0.3672	0.4	0.3607	0.5481	0.8704	0.2312	0.5532	1.369
			Neuropathy Month 2	0.3708	0.4062	0.4465	0.504	0.8613	0.2236	0.5557	1.335
			Neuropathy Month 3	0.4034	0.3704	0.3879	0.5334	1.15	0.2238	0.7414	1.782
			Neuropathy Month 4	0.3779	0.3976	0.1379	0.7104	0.9204	0.2233	0.5941	1.426

^*^Indicates a nominal significance for the marker for a phenotype at a particular timepoint

## Discussion

In a population of cancer patients, all who received CIPN-causing cancer treatment agents, were measured for CIPN using the PRO-CTCAE and were based on participants. The PRO-CTCAE has demonstrated content validity (Hay et al. 2014), construct validity, reliability, responsiveness, equivalence and acceptability across different modes of administration for a diverse range of cancer types (Bennett et al. 2016). Patient demographics and summaries of the cancer subtypes and comorbidities support that CIPN can be a result of a diverse range of cancer types and comorbidities. However, further investigation of these is necessary as it is unclear whether these may contribute to the length or severity of CIPN as this was not investigated in this study. Other studies have identified that complications related to diabetes and autoimmune diseases may increase the risk and/or severity of CIPN (Hershman et al. 2016).

The number of cases affected with CIPN in this study, *n* = 130 (75.6%) is at the higher end of the range of ~ 19–85% of patients who will exhibit CIPN by the end of treatment. The overall prevalence of CIPN at one month following the completion of chemotherapy is estimated to be around 68%, with this dropping to 60% at three months, and continuing to drop beyond six months post-chemotherapy. However, CIPN may not resolve for up to 30% of patients (Fallon 2013; Hershman et al. 2016; Seretny et al. 2014). Another consideration of this study is the low number of participants available which may have decreased our ability to accurately determine an association with these markers and overall CIPN.

Despite this, analyses were completed for an association with these markers and overall CIPN based on previous studies investigating CIPN and in relation to the *GSTT1* null allele, the *GSTM1-*null allele and *GSTP1* SNP (rs1695) (Cliff et al. 2017). This analysis failed to replicate studies which had previously identified associations with these polymorphisms and CIPN. This is consistent with an in-depth meta-analysis that had previously shown inconsistencies with identifying any genetic association and CIPN (Cliff et al. 2017). However, as this study only focused on common polymorphisms within these genes, we cannot rule out the possibility that other loss of function variants may be causative of CIPN.

Investigation of these markers in relation to pain experienced, identified that there were two markers, the *GSTM1* and *GSTT1* null mutations were significantly associated with pain at month 2 of chemotherapy. The *GSTT1* null allele showed a decreased risk (*P*-val = 0.038, OR = 0.5476), while the *GSTM1* null allele showed an increased risk (*P*-val = 0.030, OR = 1.644) for pain at month 2. It is not clear how the *GSTT1* null allele contributes to decreased risk of pain at month 2 as there is little evidence in the literature that has identified a link between *GSTT1* and pain. The pain item on the PRO-CTCAE was measured separately to the CIPN item and therefore the relationship between these two items remains unclear. However, we noted higher severity of pain in the CIPN cohort persisted at each time-point compared to those without CIPN.

In *GSTM1* null carriers, a previous study has shown the GSTM2 enzyme compensates for the lower expression and lost function of GSTM1 (Bhattacharjee et al. 2013). *GSTM2* has been linked to regulating the production of prostaglandin E2 (PGE_2_) from prostaglandin H2 PGH_2_, one of the potent mediators of pain and inflammation in the body (Trebino et al. 2003). It could be theorised that the compensatory expression of *GSTM2* in individuals with the *GSTM1* null allele may also predispose patients to increased pain through PGE_2_ production. Furthermore, PGE_2_ has also been found to be increased following chemotherapeutic treatment of Taxanes (Altorki et al. 2005). What contradicts this explanation is that the association is only evident at month 2 of chemotherapy. To date, there have been no previous associations identified between pain and *GSTM1*, however, a recent GWAS using the UK biobank data identified a locus (*KCND3* rs197422 chr1:112,000,000) that is within 1.5Mbps of *GSTM1* (GRCh38.013 chr1:109,687,817–109,693,745) as significantly associated with chronic pain (Johnston et al. 2019). This is within the estimated 2Mbp previously estimated as the causal location of an affected gene (Brodie et al. 2016).

## Conclusion

Investigation into CIPN association with polymorphisms in *GSTM1, GSTT1* and *GSTP1,* identified no significant results in a mixed cancer cohort. Despite this, associations for the *GSTM1*-null and *GSTT1-*null polymorphisms with pain at month 2 following chemotherapy were identified, however the significance of this finding remains unclear. As no significant role was identified in this cohort, we would recommend larger investigations (increased number of samples and controls) that focus on all genes, to elucidate genetic contributions to CIPN susceptibility and severity. Sequencing based studies would also be recommended, as this may provide additional information on rarer loss of function mutations within these genes contributing CIPN susceptibility and/or severity.

## Supplementary Information

Below is the link to the electronic supplementary material.

Supplementary file1 (DOCX 21 KB)

Supplementary file2 (DOCX 16 KB)

## Data Availability

All data relevant for this study is included within this manuscript. Due to the medical nature and ethical agreements for use of samples, any further information regarding the cohort may be made available on request.
