# Supplementary material for: GSTM1 and GSTT1 polymorphisms associated with pain in a chemotherapy-induced peripheral neuropathy cohort
Source: J Cancer Res Clin Oncol. 2023 Mar 20;149(10):7405–12. doi: 10.1007/s00432-023-04677-3 (PMC10374820; doi:10.1007/s00432-023-04677-3)
Supplement: Supplementary file 1 — Supplementary file1 (DOCX 21 KB) [file 432_2023_4677_MOESM1_ESM.docx]

Supplementary 1 - Protocols for PCRs and Enzyme Digestions for each gene used for this study

| Initial PCR | | | | | | |
| --- | --- | --- | --- | --- | --- | --- |
|  | ***GSTT1*** | | ***GSTM1*** | | ***GSTP1*** | |
| 5X PCR Buffer | 4.0uL |  | - |  | - |  |
| 25mM MgCl2 | 2.0uL |  | - |  | - |  |
| dNTP | 0.8uL |  | - |  | - |  |
| GSTT1 Primer Pair | 1.6uL |  | - |  | - |  |
| GSTM1 Primer Trio | - |  | 7.5uL (2.5uL each) |  | - |  |
| GSTP1 Primer Pair | - |  | - |  | 4.0uL |  |
| PCR Premix Buffer I (Epicenter) | - |  | 7.0uL |  | - |  |
| PCR Premix Buffer H (Epicener) | - |  | - |  | 7.0uL |  |
| Beta-Actin Primer Pair | 1.6uL |  | 1.0uL |  | - |  |
| Taq Flexi | 0.2uL |  | 0.2uL |  | 0.2uL |  |
| dH2O | 7.8uL |  | 7.3uL |  | 6.8uL |  |
| DNA Input (20ng/uL) | 2.0uL |  | 2.0uL |  | 2.0uL |  |
| Total Rxn | 20uL |  | 25uL |  | 20uL |  |
|  |  |  |  |  |  |  |
| Initial Denaturation | 94C for 3 mins | x1 | 94C for 4 mins | x1 | 94C for 3 mins | x1 |
| Cycle 1 Denauration | 94C for 1 min | x39 | 94C for 45 sec | x5 | 95C for 15 sec | x5 |
| Cycle 1 Annelaing | 66C for 1 min |  | 62C for 1 min |  | 64C for 30 sec |  |
| Cycle 1 Extension | 72C for 75 sec |  | 72C for 90 sec |  | 72C for 1 min |  |
| Cycle 2 Denaturation | 94C for 1 min | x1 | 94C for 30 sec | x30 | 95C for 15 sec | x25 |
| Cycle 2 Annealing | 66C for 1 min |  | 62C for 30 sec |  | 59C for 30 sec |  |
| Cycle 2 Extension | 72C for 5 mins |  | 72C for 45 sec |  | 72C for 1 min |  |
| Final Extension | - |  | 72C for 5mins |  | - |  |
| Storage | 4C | x1 | 4C | x1 | 4C | x1 |
| Electrophoresis: | 2% gel, 20 mins at 120V | | 2% gel, 20 mins at 120V |  | 2% gel, 20 mins at 120V |  |
|  |  |  |  |  |  |  |
| RFLP - Enzyme Digestion Assay | | | | | | |
|  | ***GSTT1*** | | ***GSTM1*** | | ***GSTP1*** | |
| PCR Product | - |  | 5.0uL |  | 5.0uL |  |
| CutSmart Buffer | - |  | 2.0uL |  | 2.0uL |  |
| *HaeII* (NEB) | - |  | 1.0uL |  | - |  |
| *BsmAI* (NEB) | - |  | - |  | 1.0uL |  |
| dH2O | - |  | 12.0uL |  | 12.0uL |  |
| Total | - |  | 20.0uL |  | 20.0uL |  |
|  |  |  |  |  |  |  |
| Digest: |  |  |  |  |  |  |
| Enzyme Digestion | - |  | 37C for 60mins | x1 | 55C for 60mins | x1 |
| Enzyme Deactivation | - |  | 80C for 20mins |  | - |  |
| Storage | - |  | 4C |  | 4C |  |
| Gel Electrophoresis | - |  | 4% gel 80V for 35 minutes |  | 4% gel 80V for 35 minutes |  |

Supplementary 1 Primer sequences for markers of interest. E7A – Exon 7A primer, E7B – Exon 7B prier, I6 – Intron 6, F – Forward primer, R – Reverse Primer

| **Primer** | **Sequence (5′→3′)** |
| --- | --- |
| GSTM1-E7A | TTG GGA AGG CGT CCA AGC GC |
| GSTM1-E7B | TTG GGA AGG CGT CCA AGC AG |
| GSTM1-I6 | GCT TCA CGT GTT ATG AAG GTT C |
| GSTT1-F | TTC CTT ACT GGT CCT CAC ATC TC |
| GSTT1-R | TCA CCG GAT CAT GGC CAG CA |
| β-Actin-F | CGG AAC CGC TCA TTG CC |
| β-Actin-R | ACC CAC ACT GTG CCC ATC TA |
| GSTP1-F | GTA GTT TGC CCA AGG TCA AG |
| GSTP1-R | AGC CAC CTG AGG GGT AAG |
