# Supplementary material for: GSTM1 and GSTT1 polymorphisms associated with pain in a chemotherapy-induced peripheral neuropathy cohort
Source: J Cancer Res Clin Oncol. 2023 Mar 20;149(10):7405–12. doi: 10.1007/s00432-023-04677-3 (PMC10374820; doi:10.1007/s00432-023-04677-3)
Supplement: Supplementary file 2 — Supplementary file2 (DOCX 16 KB) [file 432_2023_4677_MOESM2_ESM.docx]

Supplementary 2 Demographics related to cancer and comorbidities seen in the participant population in relation to their worst self-reported CIPN severity. *The percentage calculation for the cumulative number of samples is related to the total population of n=172 participants.

| **Worst CIPN severity rating post treatment** | **None (n=42)** | **Mild (n=50)** | **Moderate and Severe (n=80)** |
| --- | --- | --- | --- |
| Age (mean (SD)) | 62.69 (12.02) | 60.51 (13.46) | 60.51 (12.67) |
|  |  |  |  |
| **Cancer Diagnosis (%)** |  |  |  |
| Breast | 11 (26.2) | 22 (44.0) | 36 (45.0) |
| Colorectal | 3 ( 7.1) | 4 (8.0) | 10 (12.5) |
| Haematological | 2 ( 4.8) | 2 (4.0) | 4 (5.0) |
| Lung | 9 (21.4) | 8 (16.0) | 4 (5.0) |
| Other | 12 (28.6) | 9 (18.0) | 20 (25.0) |
| Unknown | 5 (11.9) | 5 (10.0) | 6 (7.5) |
|  |  |  |  |
| **Comorbidities** |  |  |  |
| heart disease (%) | 5 (11.9) | 7 (14.0) | 6 (7.5) |
| High Blood Pressure (%) | 13 (31.0) | 21 (42.0) | 21 (26.2) |
| Lung disease (%) | 6 (14.3) | 6 (12.0) | 8 (10.0) |
| Diabetes (%) | 3 ( 7.1) | 4 (8.0) | 9 (11.2) |
| Ulcer or Stomach Disease (%) | 1 ( 2.4) | 1 (2.0) | 2 (2.5) |
| Kidney Disease (%) | 1 ( 2.4) | 1 (2.0) | 2 (2.5) |
| Liver Disease (%) | 0 ( 0.0) | 2 (4.0) | 3 (3.8) |
| Anaemia or Other Blood Disease (%) | 5 (11.9) | 2 (4.0) | 8 (10.0) |
| Depression (%) | 7 (16.7) | 5 (10.0) | 8 (10.0) |
| Osteoarthritis/Degenerative Arthritis (%) | 8 (19.0) | 6 (12.0) | 12 (15.0) |
| Rheumatoid Arthritis (%) | 0 ( 0.0) | 1 (2.0) | 3 (3.8) |
| Back Pain (%) | 5 (11.9) | 13 (26.0) | 18 (22.5) |
| Other Medical Problems (%) | 8 (19.0) | 7 (14.0) | 17 (21.2) |
